# Determinants of Health Seeking Behavior among Caregivers of Infants Admitted with Acute Childhood Illnesses at Kenyatta National Hospital, Nairobi, Kenya

**DOI:** 10.1155/2018/5190287

**Published:** 2018-12-16

**Authors:** Winfred Muringi Wambui, Samuel Kimani, Eunice Odhiambo

**Affiliations:** University of Nairobi, College of Health Sciences, School of Nursing Sciences, P.O. Box 19676- 00202 Nairobi, Kenya

## Abstract

**Background:**

Poor, delayed, or inappropriate health seeking for a sick infant with acute childhood illness is associated with high morbidity/mortality. Delay in health seeking is implicated with fatal complications and prolonged hospital stay. Thus, caregivers ought to identify danger signs and promptly seek professional help for a sick infant.

**Objective:**

Establish determinants of health seeking behavior among caregivers of infants admitted with acute childhood illnesses in Kenyatta National Hospital.

**Methods:**

A mixed method cross-sectional study involving caregivers (n=130) of sick infants. Semistructured questionnaire and two focused group discussions were used to gather data on caregiver knowledge on danger signs, health care seeking options, and decision-making regarding health care seeking. Data was analyzed with SPSS V. 22.

**Results:**

Knowledge of danger signs of infancy was poor. Immediate health seeking was associated with tertiary [P=0.009] and secondary [P=0.030] education, knowledgeability on danger signs [P=0.002], and being married [P=0.019]. Respondents who resided in urban [P=0.034] or less than a kilometer [P=0.042] from a health facility sought care immediately. Those who rated services as excellent (P=0.005) and satisfactory (P=0.025) sought care promptly.

**Conclusion:**

Poor knowledge on danger signs of infancy was common among caregivers blurring the magnitude of acute illness resulting in delayed health seeking. Knowledgeability of danger signs of infancy, high educational level, and being married were associated with immediate health care seeking. Caregivers who resided in urban setting and/or near a health facility were linked to immediate health seeking. Additionally, satisfaction and perception of quality health care services were associated with immediate health seeking. Interventions with caregivers should involve capacity building through partnership with families and communities to raise awareness of danger signs of infancy. Strengthening of health care system to offer quality basic health services could improve health seeking behavior. Provision of a seamless supply system, infrastructural support, and technical support for soft skills minimize the turnaround time which is critical.

## 1. Introduction

Infant mortality and morbidity are global health problems requiring strategic policy, programming, and investments. Recently, reports indicate 75% of all under-fives deaths occurred during their first year of life [1]. Indeed, the leading medical causes of infant and child deaths are the acute childhood illnesses which include acute respiratory infections, diarrhea, malaria, and meningitis.

These illnesses cause rapid and serious physiological derangement on the baby and the time taken to seek supportive management is of essence [1].

The survival of an infant from the physical stressors associated with acute illness is dependent on identification of cues for the illness, time lag, and the decision to seek expert help by the caregiver “the so called health seeking behavior” [2, 3]. The ability to identify the danger signs of infancy depends on, among other factors, level of education, experience, parity, social support for the caregivers, and physical environment, for instance, distance to the health facility, and personnel at the health facility, among others [4, 5]. Indeed, WHO/UNICEF have recommended the need to strengthen the family ability to identify danger signs and prompt care seeking that is timely among the interventions to curb childhood illnesses. In addition, others recommended strategies including training health care professionals to educate and share health messages with mothers/caregivers on the danger signs of infancy. The strategies also include actions mothers/caregivers should take such as seeking care promptly once danger signs are detected [1].

Delay in seeking health care for a sick infant has been attributed to several factors for example, combining home remedies with conventional treatments, inability to identify life-threatening illnesses and lack of knowledge. These challenges exist against a background of undiagnosed serious life threatening illness such as diarrhea, malaria, and meningitis [1]. The results for such unverified and/or not scientifically tested interventions are catastrophic with resultant mortality and/or complications [1]. Conversely, most of infants die despite implementation of the evidence based interventions such as Integrated Management of Childhood Illnesses (IMCI) and* Malezi Bora *that put substantial premium on early diagnosis and management of infants with treatable illnesses [6]. This calls for investigations into factors that may be associated with increased infant morbidity and mortality including delayed health seeking.

Locally, at Kenyatta National Hospital (KNH), the largest referral hospital in Kenya, approximately 2000 infants were reportedly admitted with acute childhood illness in 2015 alone [7]. Among them, approximately a third died from complications [7]. The Kenyan infant mortality rate is 39 deaths per 1000 live births [5] ranking 52^nd^ globally. Shockingly, approximately 80% of child/infant deaths are reported to occur at home [6], a call for capacity building among mothers/caregivers to reverse this worrying trend. The deaths could be reduced and/or averted by seeking prompt and appropriate care for the infants, a decision directly dependent on the health seeking behavior and mothers'/caregivers practices [3, 8, 9]. For example, the WHO IMCI Package identified gaps in recognition of acute illness, and the appropriate health seeking behavior strategy emphasizes on addressing these gaps through focused interventions [1]. However, it is not clear how caregivers of infants first respond to infant ill health as well as how they seek health care for the infants when faced with acute illnesses. This study therefore sought to determine determinants of health seeking behavior among caregivers of infants with acute childhood illnesses admitted at Kenyatta National Hospital.

## 2. Materials and Methods

### 2.1. Study Setting

The study was conducted in pediatric wards of KNH, a referral and teaching public health facility in Nairobi. KNH offers both outpatient and inpatient specialty services spanning from emergency, obstetrics and gynecology, medical, surgical, oncology, and pediatrics medicine and related interventions among others. KNH has 50 inpatient wards with a total bed capacity of 2000. The pediatric department has eight inpatient wards that admit medical, orthopedic, oncology, and surgical pediatric patients. In total, the bed capacity for pediatric department is 256 which is usually overstretched to not less than 150% on a daily basis. This study involved caregivers of infants with acute childhood illness admitted in pediatric wards of KNH.

### 2.2. Study Design and Sampling

A descriptive cross-sectional mixed method study utilized both quantitative and qualitative approaches. The study involved sampled caregivers (N=130) of infants admitted between March and June 2017 with acute childhood illnesses at KNH pediatric wards. The sample size was calculated using a formula by Fishers (1990). The calculation took into considerations: the type I error which was fixed at 0.05, with a desire of a <5% chance of drawing a false-positive conclusion, as well as type II error that was set at a level of 0.20, meaning that the researcher desires a <20% chance of a false-negative conclusion. Thus, the power was set at 0.80 or 80%, representing the probability of avoiding a false-negative conclusion. Thus, the calculation established a sample size of 130 respondents who needed to have given consent and assisted in filling out the questionnaires. The admission book with all the patient's entries was used to identify the infants admitted with acute childhood illnesses in each ward. The infants who met the inclusion criteria were consecutively sampled until the required sample size was achieved. Further, the number of respondents was proportionately disaggregated into various acute illnesses the infants they took care were admitted with, namely, pneumonia (67), diarrhea (17), malaria (13), and meningitis (33), respectively. In regard to qualitative design, two focus group discussions (FGD) were conducted with a conveniently sampled group of caregivers of infants admitted with acute childhood illnesses from two pediatric wards. However, the participants who took part in responding to questionnaires were exempted from FGD. Generally, respondents consented to the study after comprehensive explanation by the researcher and subsequently assisted in filling out the questionnaires. Additionally, participants for the FGD consented to the discussion and audiorecording before the discussion could commence as well. Each FGD comprised of 8 participants which was made possible by use of predeveloped discussion guide.

### 2.3. Data Collection

Data were collected using a researcher-assisted structured questionnaire. The questionnaires were pretested among 13 respondents sampled from a pediatric ward in Mbagathi Hospital Nairobi, a lower referral public hospital that is situated about 2 kilometers from KNH, South West of Nairobi. The pretested questionnaires were structured into demographic characteristics as well as closed ended questions to capture quantitative data on caregivers' level of knowledge on danger signs in infancy, health care seeking options, and factors influencing the caregivers' decision-making regarding health care seeking. The qualitative data was collected using guided focus group discussions which were audiorecorded using digital recorder as well as hand written notes. The discussions were moderator controlled while the recorder wrote the notes and digitally recorded the discussion.

### 2.4. Data Analysis

Data were organized, screened, and checked for completeness. Thereafter coding, inputting into computer, and cross-checking against the original data set for accuracy were carried out. Data were analyzed using computer software (SPSS Ver. 22) for which descriptive and inferential statistical outputs were generated and reported appropriately. Specifically, data were descriptively analyzed into proportions and summarized in frequency tables, while the Chi-square test of independence was used to determine relationships between various variables. Further, multiple logistic regression analysis was performed to identify factors independently associated with immediate health seeking behavior among caregivers of infants. The factors that were found to have significant association with immediate health seeking behavior were considered together in a multivariable analysis. Qualitative data was transcribed, categorized, and analyzed into themes.

### 2.5. Ethical Consideration

Ethical approval for the study was obtained from Kenyatta National Hospital-University of Nairobi Ethical Review Committee (KNH-UoN ERC) (approval number P953/12/2016). Permission to access caregivers and wards was granted by the KNH Administration (Ref. KNH/PEADS- AD/48VOL.1). Both verbal and written consent were obtained from respondents after comprehensive explanation.

## 3. Results

### 3.1. Sociodemographic Characteristics of Caregivers of Infants

Most of the respondents (53.8%) were aged 21-30 years, resided in urban settings (63.1%), were married (73.8%) as well as Christians (93.8%). Regarding the level of education, 43.8% had only attained primary school education. Half (50.0%) of the caregivers were unemployed, with some reporting of being informally employed (18.5%) and students (3.8%). Economically, majority (63.8%) of the respondents earned less than 100 USD (KShs. 10,000) while 29.2% earned between 100 and 200 USD (10,000-20,000). Most (82.3%) of the respondents reported having one to three children (Table 1).

### 3.2. Health Seeking Behaviors among the Respondents

Of the respondents, 53.8% reported to have immediately taken their infant with danger signs to the hospital (Table 2). However, 22.3% first observed them to see if the condition would worsen or subside, while 22.3% bought medication from the chemist for them. Regarding advice on what action to take on a sick infant with danger signs, 38.5% obtained advice first from nurses, neighbors (19.2%), and doctors (14.6%). About a half (56.9%) of the respondents took the child to the hospital immediately after noticing danger signs. Of those who did not take the infant to the hospital, 53.6% thought the condition was mild and would improve. The results are corroborated by findings from qualitative data in the following excerpts:

“When my child had fever I removed his clothes then consulted my neighbor who advised me to wash him with warm water mixed with menthol ointment which we believe it reduces fever”(Mother to a sick son, FGD 2, Ward 3A)

“I went to the chemist explained my child's symptoms and problems and then I was advised on the medication to buy which I bought but my baby never improved” (Mother to a sick son, FGD 1, ward 3A)

“I gave painkiller first which belonged to my other child. I had been given sometime back when my first born had the same problem so that's why I decided let me use it and observe the condition hoping my child would get well” (Mother to a sick daughter, FGD 1, ward 3C)

“We had just come from upcountry Malaba (Western Kenya) where my child had started diarrhea and vomiting and my mother-inlaw had applied some local herbs believed to sooth the stomach but by the time we reached here my baby was worse” (Mother to a sick daughter, FGD 2, ward 3B)

### 3.3. Relationship between Sociodemographic Characteristics and Health Seeking among the Respondents

In the event of an infant sickness, respondents who resided in urban settings were more likely [OR=2.19; 95%CI=1.06-4.52; P=0.034] to seek health-related help immediately compared to those from rural areas. The married were more likely (61.5%) to seek help [OR=3.54; 95%CI=1.46-8.61; P=0.005] than the single (31%). In addition, respondents (66.7%) with tertiary [OR=2.96; 95%CI=1.17-7.46; P=0.022] and secondary (62.8%) [OR=2.50; 95%CI=1.11-5.63; P=0.028] education were likely to immediately seek help for their sick infant relative to those with primary education (40.4%) (Table 3).

### 3.4. Respondents' Knowledge on Danger Signs of Infancy

**Figure 1:**
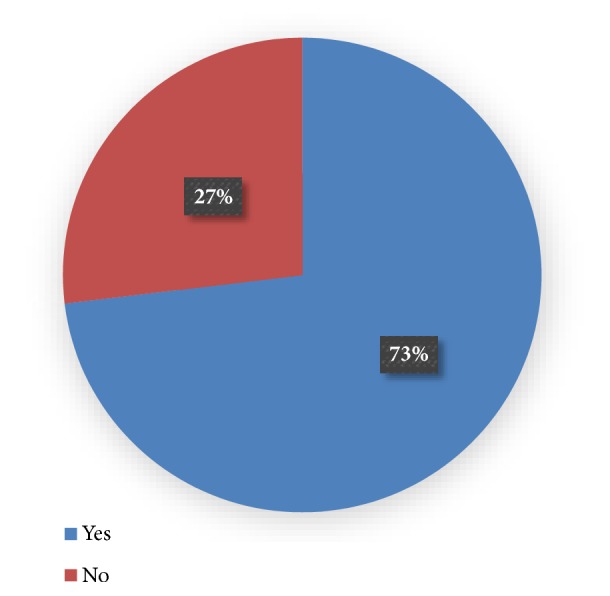
**Proportion of respondents knowledgeable with danger signs of infancy.** Most of the caregivers knew the danger signs in infancy.

**Figure 2:**
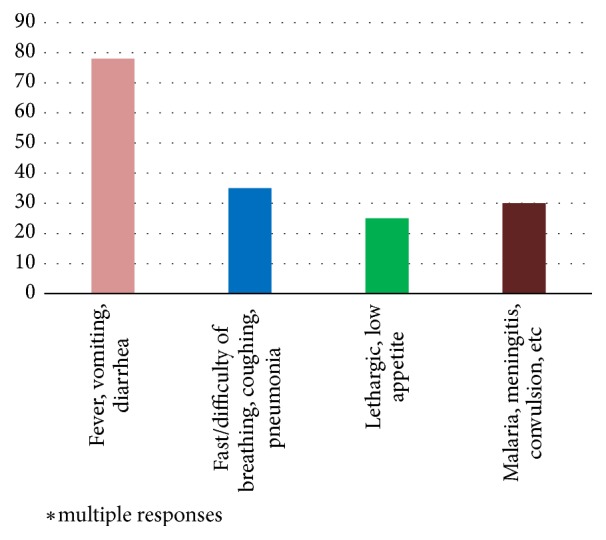
**Danger signs of infancy reported by the caregivers.** Most of the caregivers identified fever, vomiting, and diarrhea as the common danger signs of infancy.

Of the respondents, 73.1% knew of the danger signs of infancy that required immediate action. (Figure 1). The majority (60%) highlighted some of the common danger signs that included fever, vomiting, and diarrhea (Figure 2). These results are supported by findings from group discussion as shown by the following quotes:

“My child had fever, was snoring and unable to breastfeed continuously. He would breastfeed for about two minutes then stop and I would see him take a deep breath then resume breastfeeding” (Mother to a sick son, FGD 2, ward 3D)

“It started with my baby crying a lot even when I try to put her on the breast she could not stop crying. Then second day in the morning my baby suddenly started convulsing and I saw frothy saliva come out of my baby's mouth” (Mother to a sick daughter, FGD 2, ward 3C)

“The first sign I saw was that the eyes turned white, had fever and started to have diarrhea. In fact, that day I changed diapers five times and it was smelling very bad”(Mother to a sick daughter, FGD 2, ward 3D)

### 3.5. Source of Information on Danger Signs of Infancy

**Figure 3:**
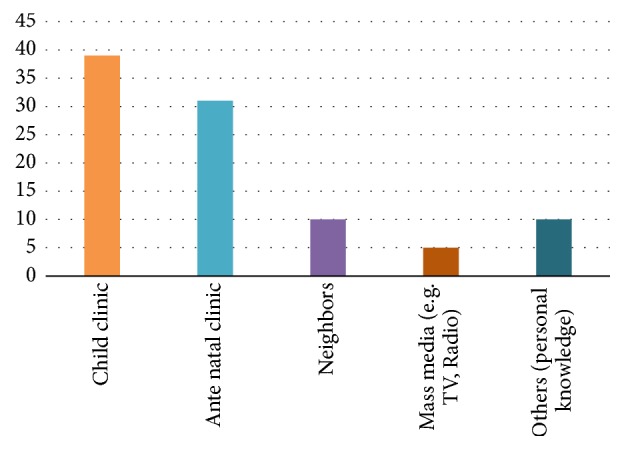
**Source of information on danger signs of infancy.** Most of the caregivers reported their main source of information on the danger signs of infancy was child clinic.

Respondents indicated the well-baby clinic (41.1%) as the most critical source of information regarding danger signs of infancy (Figure 3).

### 3.6. Relationship between Knowledge on Danger Signs and Health Seeking Behavior

Respondents who demonstrated knowledge on danger signs of infancy were more likely (65.3%) [OR=6.34; 95%CI=2.59-15.52; P=0.000] to immediately seek health-related help compared to those with low awareness (22.9%) (Table 4).

### 3.7. Relationship between Accessibility/Satisfaction and Health Seeking among Respondents

Respondents who resided less than one kilometer away from health facility were more likely (61%) [OR=2.14; 95%CI=1.02-4.48; P=0.042] to immediately seek health-related assistance compared to those staying 2KMs or more (31%). Additionally, 65.2% of respondents who rated as excellent the services offered at health facility [OR=3.75; 95%CI=1.49-9.42; P=0.005] and satisfactory (58.3%) [OR=2.80; 95%CI=1.14-6.89; P=0.025] were more likely to promptly seek health-related help relative to those who reported dissatisfaction (33.3%) (Table 5).

### 3.8. Determinants of Immediate Health Seeking for Sick Infants Admitted with Acute Childhood Illnesses

A multiple logistic regression analysis was performed to identify factors independently associated with immediate health seeking among respondents. Only three factors met the criteria to be retained during the final analysis or reduced model (Table 6). For instance, married respondents were 3.3 times more likely [AOR=3.34; 95%CI=1.22-9.18; P=0.019] to seek immediate health- related help compared to the single. Respondents with tertiary education were 4 times [OR=4.20; 95%CI=1.43-12.38; P=0.009] while those with secondary 2.8 times [AOR=2.80; 95%CI=1.11- 57.07; P=0.030] more likely to seek immediate health relative to those with primary education. In addition, those who knew the danger signs in infancy were 6.3 times [AOR=6.28; 95%CI=2.34- 16.84; P=0.002] more likely to seek immediate health-related assistance than those who were not aware.

## 4. Discussion

Our findings showed married caregivers sought immediate health for their sick child. The motivation for the married caregivers to seek early medical help results from support and push from the partner and extended family. The support is in form of emotional, financial, physical, and material that is very critical during family member sickness. The suggestion is consistent with social network and closely knit family linkages common in African cultures where a family member is considered as part of the larger community playing critical role in health care decisions. Additionally, men are the family head, principle caregivers, and decision-makers and provide the financial resources to actualize the plans [10, 11]. Thus, with responsibilities and resources bestowed on men, they would promptly make decision to seek help for their sick infant without delay [10]. Additionally, there would be immense blame from the family and the community if something drastic happens to the sick child. In this situation, the most culpable person would always be the man, thus the need to make decisions quickly for health seeking. In contrast, gender bias and discrimination are associated with women being deprived off power or role in decision-making resulting to underutilization of health care as well as poor access to health care services [12, 13].

Importantly, caregivers residing in urban settings as well as those with secondary and/or tertiary level education sought immediate help for the sick child compared to those from rural areas. This is attributed to increased awareness via easily available media for dissemination of health information, namely, television, radio, newspaper, and proximity to well informed neighborhood. Similarly, high educational level is a maker of social economic status enabling them to read, access information and expert opinion. The findings are consistent with reports that caregivers who have attained secondary education are more knowledgeable on, common health conditions/problems and how to deal with the challenges, thus increasing the chance to seek appropriate health behavior [14]. Similarly, highly educated caregivers are better in understanding the shared health information thus making them seek care for their children without delay [10]. Other reports have adduced that mothers who are educated are more likely to make decision to seek for quality health care services, have better access to health service information, and have an improved perception of the danger signs [15].

Interestingly, although the age of caregivers did not show any significant association with immediate health seeking behavior, a considerable proportion aged 31years and above did not seek immediate help for their sick children. This underscores the role of experience in health seeking behavior which may produce pseudo-experts sometime not so safe. This is consistent with findings among caregivers aged 35 years and above from Tanzania who did not perceive diarrhea as an illness and thus were less likely to seek immediate help in case their children developed such problems [16]. Such children are more likely to develop complications like diarrhea-related shock increasing their mortality. On the other hand, young mothers were more likely to seek health- related help immediately following their infants' illness. This is attributed to lack of experience on the actions to take, thus seeking professional help. Additionally, the young mothers especially the first timers are likely to be apprehensive and more worried of losing their first child to an illness because of stigmatization and sanctions associated with family and community members. Fear of the sanctions could motivate them to seek professional help without delay once they notice the danger signs. The young mothers are also reportedly more exposed to mass media because of their level of education and this has been seen to contribute to their better health care seeking [17].

The caregivers who demonstrated knowledge on danger signs of infancy sought immediate help for their sick infant. This underscores the critical role knowledge plays and the need to build capacity for caregivers to identify danger signs in a child as well as actions they should undertake. The proposal is in line with the strategic objective of IMCI notably; to reduce the under-5 child mortality, education of the mother, and/or caregiver on home care of the child during illness [1] is important. The strategy helps in bridging the knowledge gap on identification of the danger signs for common illnesses, proper treatment, and lack of and/or limited access to the health care facilities among mothers in low income countries [18]. In our study for example, some mothers highlighted “pneumonia” and “malaria” as the danger signs but were not aware of the specific signs. This is contrary to findings from Nigeria, where mothers identified fever as the only danger sign [19]. Thus, poor caregivers' knowledge contributes to delay in seeking health for a sick infant. Indeed, evidence shows that caregivers' awareness of danger signs actually promotes health seeking behavior [11]. Such awareness can be promoted and improved through introduction of community-based IMCI programs [20].

Substantial number of caregivers lived near health care facility with some reporting the services offered were unsatisfactory. Their dissatisfaction was based on the following: very slow pace of services, poor laboratory services, and lack of supplies; medicine, equipment, and lack of proper assessment of children. Some of the reasons can be attributed to shortage of staff, infrastructural challenges, and poor financing for the health care system. However, the reasons are associated with poor health seeking behavior and may contribute to complications as well as high mortality. On the other hand, caregivers who rated services offered in health facilities as excellent sought immediate health care. This implies that satisfaction is a determinant of immediate health care seeking during acute childhood illnesses. Thus, staffing, infrastructure, and health financing are critical factors that can influence health seeking behavior; thus policy-makers, programmers should aim to address the aforementioned.

Health seeking behavior is inversely related to the distance to health facility. The caregivers who stayed less than a kilometer from the health care facility were more likely to seek immediate help for their sick infant. The distance is attributed to added cost of transport as well as lack of interaction with the facility by the community members. These findings are consistent with reports of significant reduction in the utilization of health care services because the distance from a health facility increased [21]. Furthermore, the findings concur with those of Kante and colleagues who found children of caregivers who lived near the health facility were likely to receive treatment for the illnesses compared to those from far [16].

The severity of the illness determines the action to be taken by the caregivers. Caregivers seek health care when they perceived that the illness is severe and they do not see the reason for seeking health care if the illnesses is mild or the illness is not for medical attention [14]. Caregivers in our study who reported not taking their children to the hospital immediately after noticing danger signs thought the illness was mild and would improve. It is possible that most caregivers delay seeking health care when they perceive symptoms as mild, which causes delays in seeking medical care risking complications and mortality. This closely agrees with other studies where majority of the caretakers considered diarrhea to be a mild illness that does not warrant a visit to care provider outside home [14, 22].

There was no association between socioeconomic characteristics and health seeking behavior. However, most of the respondents mentioned lack of finance as the main hindrance in seeking health care outside their home, those who earned less delayed seeking medical assistance for their sick infant. Poor economic status has influence on the respondents' health seeking behavior. This is consistence with findings that low social economic status has been associated with poor health seeking behaviors and poor utilization of health care facilities [23]. People with low socioeconomic status are often unable to afford health services due to the high cost. Once socioeconomic status of the urban poor improves, they may overcome financial constraints thus seeking care immediately and promptly for a child with childhood illness [20].

Our study holds a number of limitations. Health seeking behavior was evaluated cross-sectionally by asking what might have happened in the past. Thus recall bias may not have been completely eliminated, although such bias may not have been substantial. This was a cross-sectional descriptive study involving a sample size of 130 respondents. Because of the design and small sample size, the causal relationship between characteristics and health seeking may not be strongly established. In addition, the small sample size may not have power to allow for statistical difference between some variables.

## 5. Conclusion

In conclusion, Poor knowledge on danger signs of infancy was common among caregivers blurring the magnitude of acute illness resulting in delayed health seeking. Knowledgeability of danger signs of infancy, high educational level, and being married were associated with immediate health care seeking. Caregivers who resided in urban setting and/or near a health facility were linked to immediate health seeking. Additionally, satisfaction and perception of quality health care services were associated with immediate health seeking. Interventions with caregivers should involve capacity building through partnership with families and communities to raise awareness of danger signs of infancy. Strengthening of health care system to offer quality basic health services could improve health seeking behavior. Provision of a seamless supply system, infrastructural support, and technical support for soft skills minimize the turnaround time which is critical.

## Figures and Tables

**Table 1:** Sociodemographic characteristics of the respondents.

**Characteristics**	**Frequency (n=130)**	**Percent (**%**)**
**Age in years**		
11-20	11	8.5
21-30	70	53.8
31-40	46	35.4
41-50	2	1.5
51 and above	1	0.8
**Residence**		
Rural	48	36.9
Urban	82	63.1
**Marital status**		
Single	29	22.3
Married	96	73.8
Separated/widowed	5	3.8
**Religion**		
Christian	122	93.8
Muslim	7	5.4
Pagan	1	0.8
**Level of education**		
Primary	57	43.8
Secondary	43	33.1
College	30	23.1
**Occupation**		
Self-employed	36	27.7
In formal employment	24	18.5
Not-employed	65	50
Student	5	3.8
**Gross household income per month in Kenyan shillings**		
<10,000	83	63.8
10,000-20,000	38	29.2
20,000-50,000	8	6.2
>50,000	1	0.8
**Number of children**		
1 to 3	107	82.3
4 to 6	18	13.8
7 to 9	2	1.5
10 or more	3	2.3

**Table 2:** Actions related to health seeking among respondents.

**Actions related to health seeking**	**Frequency (n=130)**	**Percent (**%**)**
**First action to be taken when danger signs in the child are identified**		

Took the child to the hospital immediately	70	53.8

Observed to see if it will worsen	29	22.3

Bought medication from the chemist	29	22.3

Used home remedies	2	1.5

**From whom do you get advice first when you identify a danger sign in your child**		

Nurse	50	38.5

Neighbors	25	19.2

Doctor	19	14.6

My husband	13	10

In-laws (mother- in- law, father- in- law)	12	9.2

Grandparents of the child	8	6.2

Traditional healers	3	2.3

**Reasons for choice of advice**		

They know more and are professional	56	60.2

They are closer or we live together	21	22.6

They have children too	9	9.7

They have children too	9	9.7

Missing	37	

**Duration to take the child to the Hospital after noticing danger signs**		

Immediately	74	56.9

1-2 days	35	26.9

More than 2 days	21	16.2

**Reasons for not immediately taken to hospital**		

It was mild and thought it would improve	30	53.6

Gave calpol, home remedies or medication and thought the baby would improve	10	17.9

Thought it was teething	8	14.3

Others	5	8.9

Money problem	3	5.4

Not applicable	74	

**Table 3:** Relationship between sociodemographic characteristics and health seeking among the respondents.

**Variables**	**Immediate health seeking behavior**				**OR**	**95**%** CI**		**p value** *∗*
	**Yes**		**No**					
	**N**	%	**n**	%		**Lower**	**Upper**	
**Age in years**								

11-20	6	54.5	5	45.5	1.25	0.34	4.64	0.739

21-30	40	57.1	30	42.9	1.39	0.67	2.89	0.380

31 and above	24	49.0	25	51.0	Ref			

**Residence**								

Rural	20	41.7	28	58.3	Ref			

Urban	50	61.0	32	39.0	2.19	1.06	4.52	**0.034**

**Marital status**								

Single	9	31.0	20	69.0	Ref			

Married	59	61.5	37	38.5	3.54	1.46	8.61	**0.005**

Separated/widowed	2	40.0	3	60.0	1.48	0.21	10.46	0.693

**Religion**								

Christian	66	54.1	56	45.9	1.57	0.34	7.32	0.562

Muslim	3	42.9	4	57.1	Ref			

**Level of education**								

Primary	23	40.4	34	59.6	Ref			

Secondary	27	62.8	16	37.2	2.50	1.11	5.63	**0.028**

College	20	66.7	10	33.3	2.96	1.17	7.46	**0.022**

**Number of children**								

1 to 3	58	54.2	49	45.8	Ref			

4 to 6	9	50.0	9	50.0	0.85	0.31	2.30	0.741

7 and above	3	60.0	2	40.0	1.27	0.20	7.89	0.800

^*∗*^Significant at p<0.05 bolded; OR= odds ratio; CI= confidence interval; Ref = reference.

**Table 4:** Relationship between knowledge on danger signs and health seeking behavior.

**Variables**	**Immediate health seeking**		**No immediate health seeking**		**OR**	**95**%** CI**		**p value** *∗*
	**N**	%	**N**	%		**Lower**	**Upper**	
**Whether familiar with danger signs in infancy**								
Yes	62	65.3	33	34.7	6.34	2.59	15.52	**0.000**
No	8	22.9	27	77.1	Ref			

^*∗*^Significant at p<0.05 bolded; OR= odds ratio; CI= confidence interval; Ref = reference.

**Table 5:** Relationship between accessibility/satisfaction and health seeking among respondents.

**Variables**	**Immediate health seeking**		**No immediate health seeking**		**OR**	**95**%** CI**		**p value** *∗*
	**n**	%	**N**	%		**Lower**	**Upper**	
**Any health facility near where you live**								
Yes	70	55.1	57	44.9	Ref			
No	0	0.0	3	100.0	UD	UD	UD	0.096
**Type of health facility near where you live**								
Dispensary	16	55.2	13	44.8	1.44	0.50	4.16	0.505
Health center	34	59.6	23	40.4	1.73	0.68	4.39	0.253
Hospital (Sub-county, county and national referral)	8	53.3	7	46.7	1.33	0.37	4.77	0.658
Private clinic	12	46.2	14	53.8	Ref			
**Distance of the nearest health facility**								
Less than 1km	50	61.0	32	39.0	2.14	1.02	4.48	**0.042**
More than 2kms	19	42.2	26	57.8	Ref			
**Rating the services offered in health facility**								
Excellent	30	65.2	16	34.8	3.75	1.49	9.42	**0.005**
Satisfactory	28	58.3	20	41.7	2.80	1.14	6.89	**0.025**
Not satisfactory	12	33.3	24	66.7	Ref			

^*∗*^Significant at p<0.05 bolded; OR= odds ratio; CI= confidence interval; Ref = reference; UD= undefined.

**Table 6:** Determinants of immediate health seeking for sick infants admitted with acute childhood illnesses.

**Variables**	**AOR**	**95**%** CI**		**p value** *∗*
		**Lower**	**Upper**	
Full model				

**Residence**				

Rural	Ref			

Urban	1.11	0.43	2.91	0.829

**Marital status**				

Single	Ref			

Married	3.33	1.10	10.06	**0.033**

Separated/widowed	1.31	0.15	11.68	0.810

**Level of education**				

Primary	Ref			

Secondary	2.66	1.02	6.89	**0.044**

College	3.53	1.09	11.37	**0.035**

**Whether familiar with danger signs in infancy**				

Yes	5.22	1.88	14.47	**0.002**

No	Ref			

**Distance of the nearest health facility**				

Less than 1km	1.95	0.80	4.76	0.140

More than 2kms	Ref			

**Rating the services offered in health facility**				

Excellent	1.93	0.65	5.74	0.234

Satisfactory	1.44	0.50	4.14	0.498

Not satisfactory	Ref			

Reduced model				

**Marital status**				

Single	Ref			

Married	3.34	1.22	9.18	**0.019**

Separated/widowed	1.35	0.15	11.79	0.788

**Level of education**				

Primary	Ref			

Secondary	2.80	1.11	7.07	**0.030**

College	4.20	1.43	12.38	**0.009**

**Whether familiar with danger signs in infancy**				

Yes	6.28	2.34	16.84	**0.000**

No	Ref			

AOR = adjusted odds ratio; CI = confidence interval; Ref = reference; *∗*significant p value bolded.

## Data Availability

The data used to support the findings of this study are available from the corresponding author upon request.
